# Chromosome X aneusomy and androgen receptor gene copy number aberrations in apocrine carcinoma of the breast

**DOI:** 10.1007/s00428-021-03028-2

**Published:** 2021-02-03

**Authors:** Anna Cremonini, Luca Saragoni, Luca Morandi, Angelo G. Corradini, Caterina Ravaioli, Enrico Di Oto, Francesco Limarzi, Alejandro M. Sanchez, Maria C. Cucchi, Riccardo Masetti, Cecily Quinn, Maria P. Foschini

**Affiliations:** 1 Anatomic Pathology Section, Department of Oncology, Bellaria Hospital, AUSL Bologna, Via Altura 3, 40139 Bologna, Italy; 2 Pathology Unit, Morgagni-Pierantoni Hospital, Forlì, Italy; 3 Department of Biomedical and Neuromotor Sciences, Functional MR Unit, IRCCS Istituto delle Scienze Neurologiche di Bologna, University of Bologna, 40139 Bologna, Italy; 4 Anatomic Pathology Section “M. Malpighi” Department of Biomedical and Neuromotor Sciences, University of Bologna, 40139 Bologna, Italy; 5 Laboratory of Molecular Pathology and Anatomic Pathology, S. Orsola Clinical Hospital, Viale Ercolani 4/2, 40138 Bologna, Italy; 6 Multidisciplinary Breast Center – Dipartimento Scienze della Salute della donna e del Bambino e di Sanità Pubblica, Fondazione Policlinico Universitario A. Gemelli IRCCS, 00168 Rome, Italy; 7 Unit of Breast Surgery, Department of Oncology, Bellaria Hospital, AUSL Bologna, 40139 Bologna, Italy; 8 Department of Histopathology, St. Vincent’s University Hospital, Dublin, Ireland; 9 School of Medicine, University College Dublin, Dublin, Ireland

**Keywords:** Carcinoma with apocrine differentiation, Androgen receptor, Triple negative breast cancer, DNA methylation, X chromosome

## Abstract

Carcinomas with apocrine differentiation (CAD) of the breast are rare tumours typically presenting high immunohistochemical expression of androgen receptor (AR) which is a target molecule for personalised therapy. To date, no studies have evaluated the genetic changes that are associated with AR immunohistochemical expression in CADs. The present work aims to characterise AR status in CADs. Twenty CAD tumours were studied with immunohistochemistry, in situ fluorescence hybridization and DNA methylation analysis, to evaluate AR expression and its regulator status. All tumours demonstrated high AR immunohistochemical expression, with over 95% of the neoplastic cells showing AR positivity in 19/20 cases. CADs showed AR gene copy loss in a percentage of neoplastic cells ranging from 5 to 84% (mean 48.93%). AR regulator genes, including the *MAGE* family, *UXT* and *FLNA*, presented variable methylation levels, but were mainly hypomethylated and therefore all transcriptionally active. The results of this study indicate that CADs present AR monosomy, paralleled by higher transcriptional activity of the gene with potential to influence response to AR deprivation therapy.

**Supplementary Information:**

The online version contains supplementary material available at 10.1007/s00428-021-03028-2.

## Introduction

In the study of oestrogen (ER) and progesterone (PR) receptor-negative tumours, attention is currently focused on cases that express androgen receptor (AR). In most of the cases, these tumours display the morphological features of carcinoma with apocrine differentiation (CAD) of the breast (1), composed of large cells with abundant, granular and eosinophilic cytoplasm, a centrally located nucleus with a thick nuclear membrane, coarse nuclear chromatin and a prominent nucleolus [1–3]. Gross cystic disease fluid protein 15 (GCDFP15) is frequently positive [1–3]. CADs are generally triple negative or show *HER2* amplification, in addition to strong and diffuse AR positivity on immunohistochemistry [1–3]. AR is a targetable molecule as has been demonstrated mainly in prostate [4] and, more recently, in male breast cancer [5]. Trials have been approved to evaluate anti-AR therapy efficacy in women affected by AR-positive tumours with promising results [4].

To date, most studies of CADs are based on immunohistochemical evaluation of AR expression in neoplastic cells. The AR gene is located on X chromosome at Xq11–12 [6, 7]. In addition to AR, a group of genes involved in the regulation of AR function and expression are located on X chromosome: *FLNA*, *UXT* and the *MAGE* family genes, (*MAGEA1*, *MAGEA2*, *MAGEA3*, *MAGEA9*, *MAGEA11*, *MAGEC1*, *MAGEC2*) [8]. Recent studies have demonstrated a role for AR in the neoplastic transformation of male breast [9]. In previous studies performed at our institution, it has been demonstrated that neoplastic cells in male breast cancer acquire additional copies of X chromosome with consequent AR polysomy [10, 11]. There are currently no data on X chromosome and AR copy number variations in female breast CADs. The aim of this study was to evaluate the AR status, X chromosome copy number variations and the methylation pattern of AR regulators in a series of CADs.

## Materials and methods

### Case selection

Consecutive cases were retrieved from the files of the Surgical Pathology Units of the Department of Biomedical and Neuromotor Sciences at Bellaria Hospital in Bologna (Italy), St. Vincent’s University Hospital in Dublin (Ireland) and Morgagni Hospital in Forlì (Italy).

### Selection criteria

Cases were retained for the present study when they showed (a) ER and PR negativity and AR positivity; (b) the morphological and immunohistochemical profile consistent with the diagnosis of CAD according to recent guidelines [1]; (c) sufficient tissue to perform in situ hybridization and molecular tests. All cases had been diagnosed in the period January 2000–December 2016. All cases with their immunohistochemical profile were reviewed, classified and graded according to currently available criteria [1]. All tissue specimens had been fixed in buffered formalin for 24 h and then paraffin-embedded (FFPE) according to routine procedures.

### Immunohistochemistry (IHC)

Immunohistochemistry was performed on an automated stainer (Ventana BenchMark, Ventana Medical Systems Inc., Tucson, AZ, USA) applying a pre-diluted monoclonal anti-androgen receptor (Cell Marque, clone SP 107) and anti-GCDFP-15 (Thermo Fisher Scientific, clone 23A3) antibodies.

### Fluorescent in situ hybridization (FISH)

**Fig. 1:**
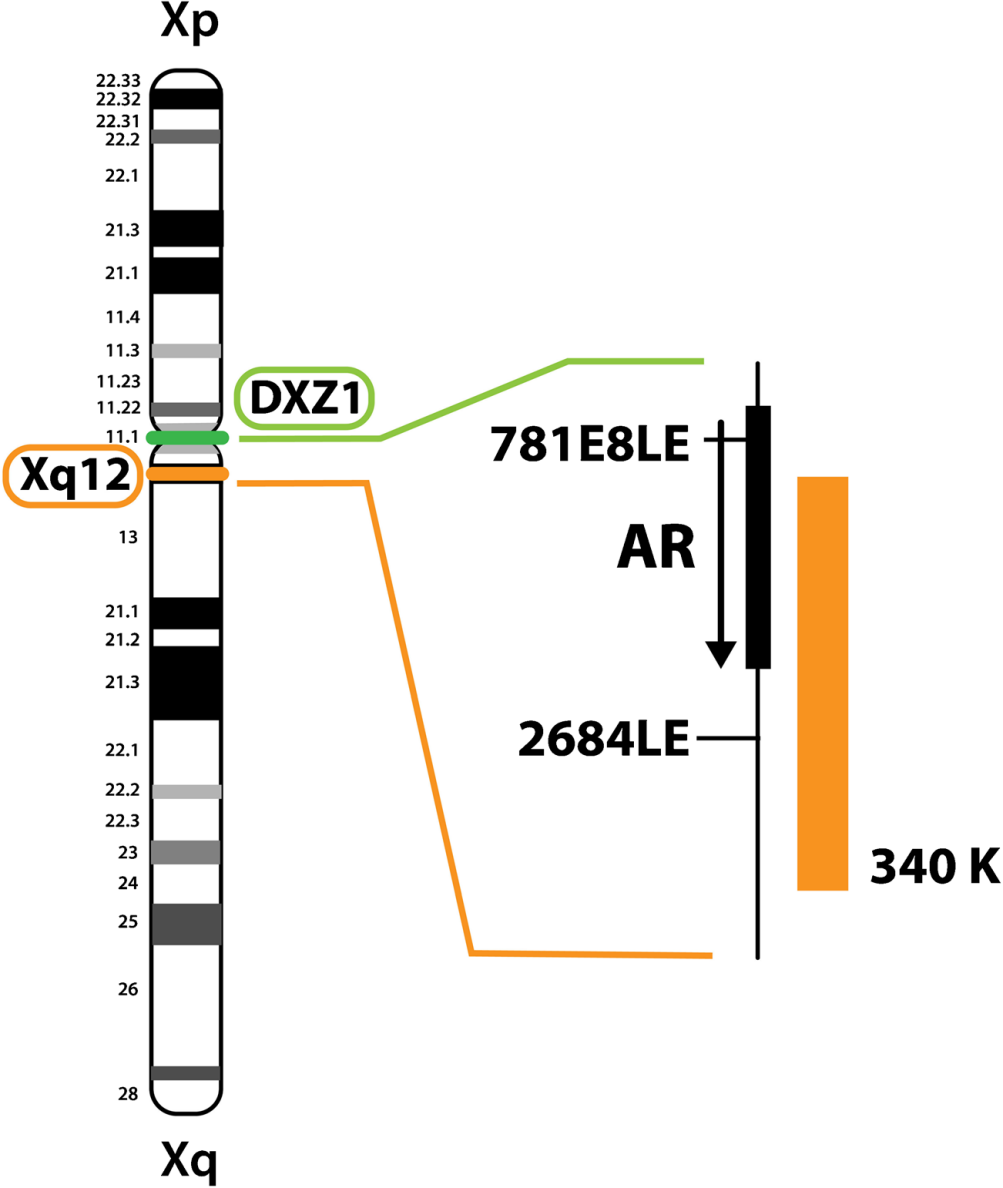
Schematic representation of X chromosomes and the anatomical location of both regions tested with FISH

Dual-colour FISH was carried out according to a standard protocol as previously described [10, 11], shortly summarized as follows: Five micron sections were obtained from each tumour block. One specific probe kit for the X chromosome (ON AR (Xq12)/SE X, Kreatech Diagnostics, Amsterdam, The Netherlands), added with the Smart-ISH hybridization buffer (OACP IE LTD, Cork, IE), was applied. The AR gene-specific probe length is stated of 340Kb (product-specific datasheet from Kreatech Diagnostics). The X chromosome centromeric region-specific probe length has not been disclosed by the producer. The two regions tested are schematically illustrated in Fig. 1.

FISH for HER2 gene amplification status was routinely performed, at the time of diagnosis, applying specific probes (ON HER2/CE 17, Kreatech Diagnostics, Amsterdam, The Netherlands) with the same method cited above.

### Evaluation criteria and data analysis

FISH analysis was carried out using an Olympus BX61 epifluorescence microscope (Olympus, Melville, NY) equipped with a 100-planar objective. For each case, a minimum of 60 non-overlapping nuclei was scored. Scoring was performed as previously described [10–12]: total number of X chromosome centromeric probe signals (spectrum green), average number of green signals, total number of AR signals (spectrum orange), average number of orange signals and ratio between green (X chromosome) and orange (AR) signals. In the non-neoplastic ductal cells, utilized as internal control, two signals for both the AR gene (spectrum orange) and the centromeric X chromosome region (spectrum green) identified the normal chromosome asset.

HER2 results were scored following currently available guidelines [13].

### Methylation analysis

Methylation analysis was performed as previously described [11]. Briefly, DNA obtained from formalin-fixed paraffin-embedded tissues was purified using the QuickExtract™ FFPE DNA extraction kit (Epicentre, Madison, WI). Two hundred to 500 ng of genomic DNA underwent bisulfite treatment applying the EZ DNA Methylation-Lightning™ Kit (Zymo Research Europe, Freiberg, Germany) according to the manufacturer’s protocol. DNA methylation was analysed by bisulfite next-generation sequencing (bisulfite-SEQ) following an internal protocol [14] using a two-step library preparation approach: the first step comprises multiplex PCR amplification for target enrichment and a second PCR amplification for specimen barcoding (primers and region details are available on Supplementary Table 1). Libraries were loaded on the MiSeq (Illumina, San Diego, CA) according to the manufacturer’s protocol. Each NGS experiment was designed to allocate 1,000 reads for any region of interest, in order to have a depth of coverage of at least 1000×. FASTQ files were processed for quality control (> Q30), reads length (> 100 bp) and converted into FASTA format in galaxy project environment [14]. In order to evaluate the methylation ratio of each CpG, a single specific file for every case and every gene was created by Perl, which was then visualized using KISMETH [15]. In parallel, reads were mapped by BWAmeth generating a bam file which was then processed by MethylDackel using hg38 as a reference; this tool created an excel file assigning at each CpG position the exact methylation level.

### Statistical analyses

Statistical analyses were performed using commercially available software: QuickCalcs, which is an online tool for linear regression by GraphPad, and Excel, from Microsoft Corporation.

The relationship between FISH results and clinical and histopathological data was calculated using the generalized Fisher’s exact test that is part of the GraphPad QuickCalcs online tool. *P* values smaller than 0.05 were considered to reflect a significant difference between groups.

### Ethical statement

All investigations were conducted according to the principles expressed in the Declaration of Helsinki; the study was approved by the local Ethical Committee (Code: n. CE17133).

## Results

**Fig. 2:**
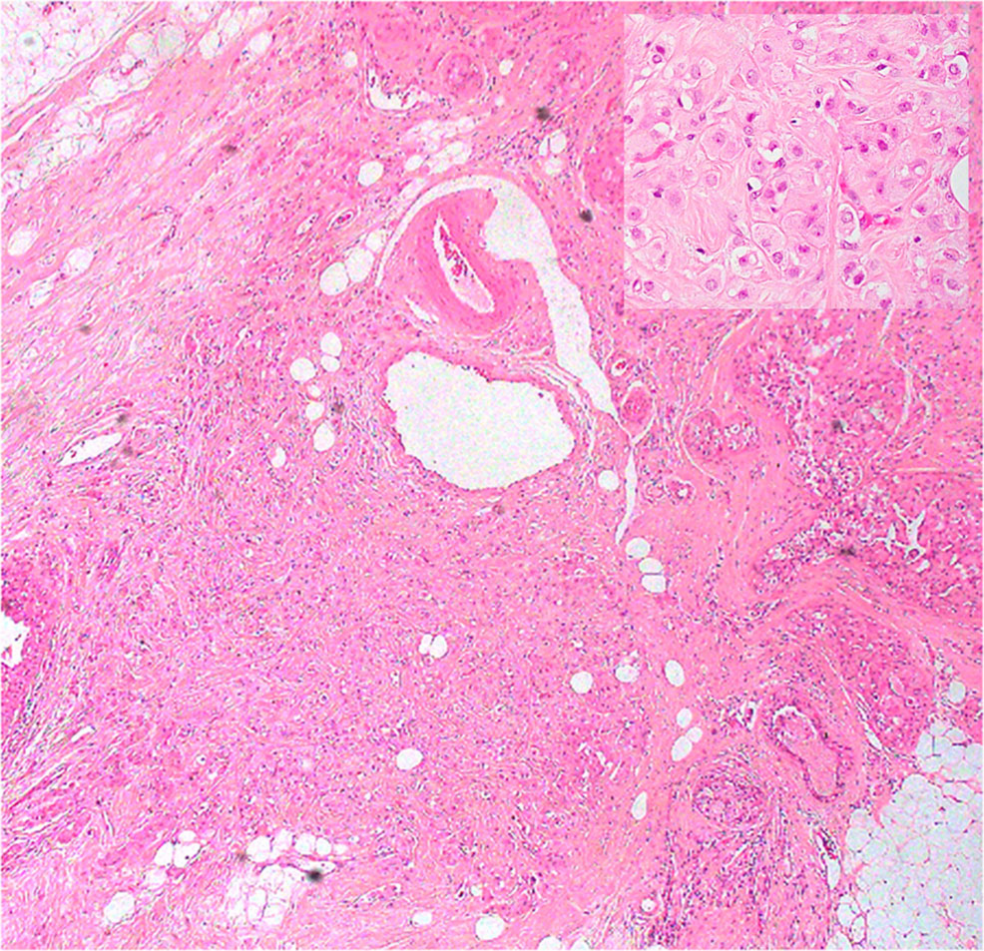
On histology, all the tumors showed the typical CAD features, being composed of eosinophilic cells. Inset: at higher power, the neoplastic cells show eosinophilic and granular cytoplasm, atypical nucleus and prominent nucleolus

**Fig. 3:**
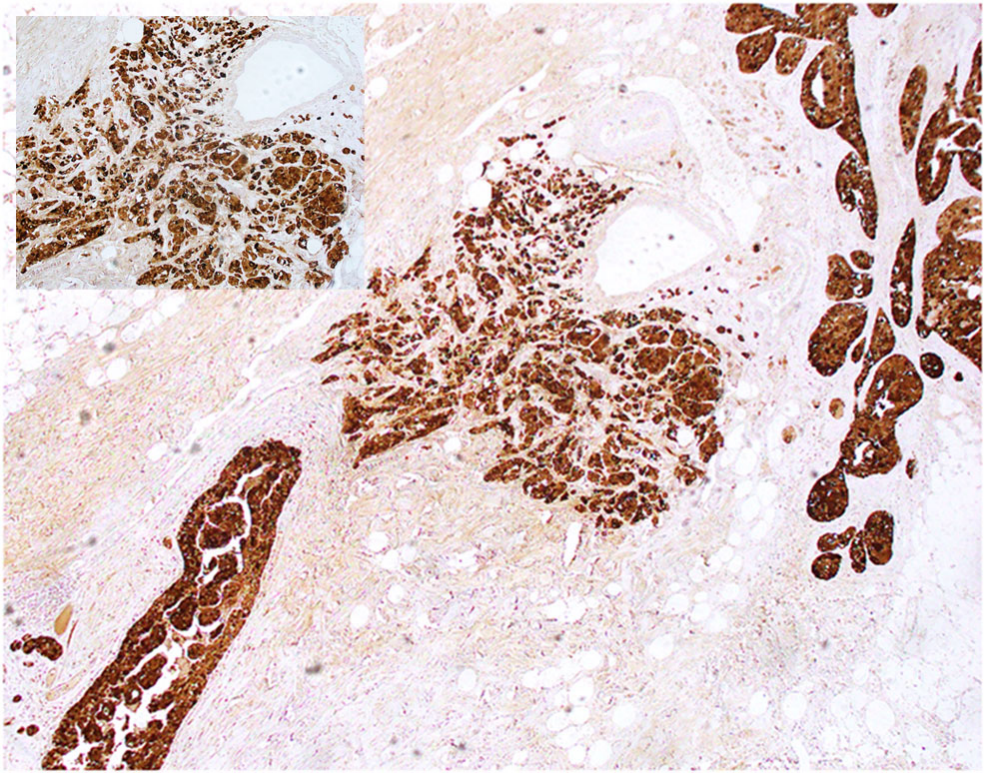
Gross cystic disease fluid protein 15 was strongly positive in most of the neoplastic cells. Inset: detail at high power

Twenty tumours met the inclusion criteria and constituted the basis of the present study. Clinical and pathological features are summarized in Table 1 (Figs. 2 and 3).

**Table 1:** Clinical and pathological details

Case	Age	Site	Size (mm)	Grade	T	N	GCDFP15	HER2	AR	ER/PR
1	58	UOQ-R	34	2	pT2	N3	P	FISH neg	+++	-
2	61	IOQ-R	16	2	pT1c	N0	P	FISH neg	+++	-
3	53	IOQ-R	2	3	pT1a	N1	P	FISH pos	+++	-
4	64	IOQ-R	13	2	pT1c	N0	P	FISH neg	+++	-
5	85	UOQ-L	30	3	pT2	N2	P	FISH neg	+++	-
5	63	IEQ-R	2	3	pT1a	N1	P	FISH pos	+++	-
7	59	RAR-R	10	2	pT1b	N0	P	FISH neg	++	-
8	57	IOQ-L	16	3	pT1c	N0	P	FISH pos	+++	-
9	49	UOQ-R	9	2	pT1b	N0	P	FISH neg	+++	-
10	58	IOQ-L	35	2	pT2	N0	P	FISH neg	+++	-
11	67	UQ-R	20	3	pT1c	N0	P	FISH pos	+++	-
12	59	R	22	3	pT2	N3	P	FISH neg	+++	-
13	56	UOQ-L	33	3	pT2	N1	P	FISH pos	+++	-
14	63	L	12	2	pT1c	N0	P	FISH neg	+++	-
15	68	R	8	3	pT1b	N0	P	FISH pos	+++	-
16	69	RAR L	9	3	pT1b	N3a	P	FISH neg	+++	-
17	77	UQ-R	42	3	pT4b	N3a	P	FISH neg	+++	-
18	83	R	10	3	pT2	N1	P	FISH pos	+++	-
19	69	UOQ-L	10	3	pT1b	N0	P	FISH pos	+++	-
20	63	UOQ-R	60	3	pT3	N3a	P	FISH neg	+++	-

*L*, left breast; *R*, right breast; *UOQ*, upper outer quadrant; *IOQ*, inner outer quadrant; *RAR*, retro-areolar region; *IEQ*, inferior external quadrant; *UQ*, upper quadrants; *N*, negative; *P*, positive; *FISH pos*, amplified on FISH analysis; *FISH neg*, not amplified on FISH analysis. +: positive in 10–25% of the neoplastic cells; ++: positive in > 50% < 94% of the neoplastic cells; +++: positive in > 95% of the neoplastic cells. *ER*, oestrogen receptor; *PR*, progesterone receptor

All patients were female, aged 49 to 85 years (mean age 64 years). Tumours were graded as grade 3 (*n* = 16) and grade 2 (*n* = 4) [16]. Nine patients had axillary lymph node metastases at the time of presentation. Invasive carcinoma size ranged from 2 to 60 mm with an average of 19.6 mm.

pTNM [17] was as follows: pT1a in 2, pT1b in 5, pT1c in 5, pT2 in 6, pT3 in 1 and pT4b in 1.

**Fig. 4:**
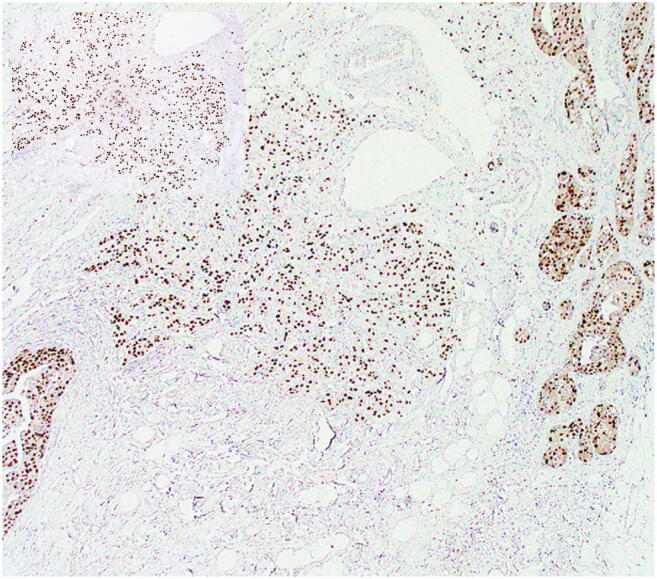
On immunohistochemistry, AR stained positive in most of the neoplastic cells. Inset: detail at higher power

In 19/20 tumours, AR stained more than 95% of the neoplastic cells (Fig. 4). In the remaining case, AR positivity was detected in 50% of the neoplastic cells (case 7).

HER2 was 3+ or amplified by FISH in 8 cases (40%).

### FISH results (Table 2, Fig. 5)

FISH analysis of the X chromosome was performed on all tumours included in the study and informative results were obtained in 13 tumours. In all cases, normal ducts served as an internal control. Tumours were eliminated from the study when the two signals (red and green) were not clearly evaluable.

**Table 2:** FISH results

Case	% nuclei AR monosomy	% nuclei XX normal asset	% nuclei X polysomy	% nuclei AR deletion	% nuclei AR single copy
1	48.57%	51.42%	0.00%	0.00%	48.57%
3	0.00%	42.85%	14.28%	42.85%	42.85%
4	25.19%	51.14%	23.66%	0.00%	25.19%
6	51.00%	16.00%	0.00%	33.00%	84.00%
7	34.88%	39.53%	0.00%	25.58%	60.46%
8	6.74%	37.07%	0.00%	56.17%	62.91%
10	65.78%	26.31%	7.89%	0.00%	65.78%
11	5.00%	95.00%	0.00%	0.00%	5.00%
12	4.76%	88.78%	0.00%	6.54%	11.21%
13	20.00%	60.00%	0.00%	20.00%	40.00%
14	6.12%	42.85%	0.00%	51.02%	57.14%
16	37.50%	42.50%	0.00%	20.00%	57.50%
19	73.10%	24.39%	0.00%	2.43%	75.53%

*X*, X chromosome; *AR*, androgen receptor

**Fig. 5:**
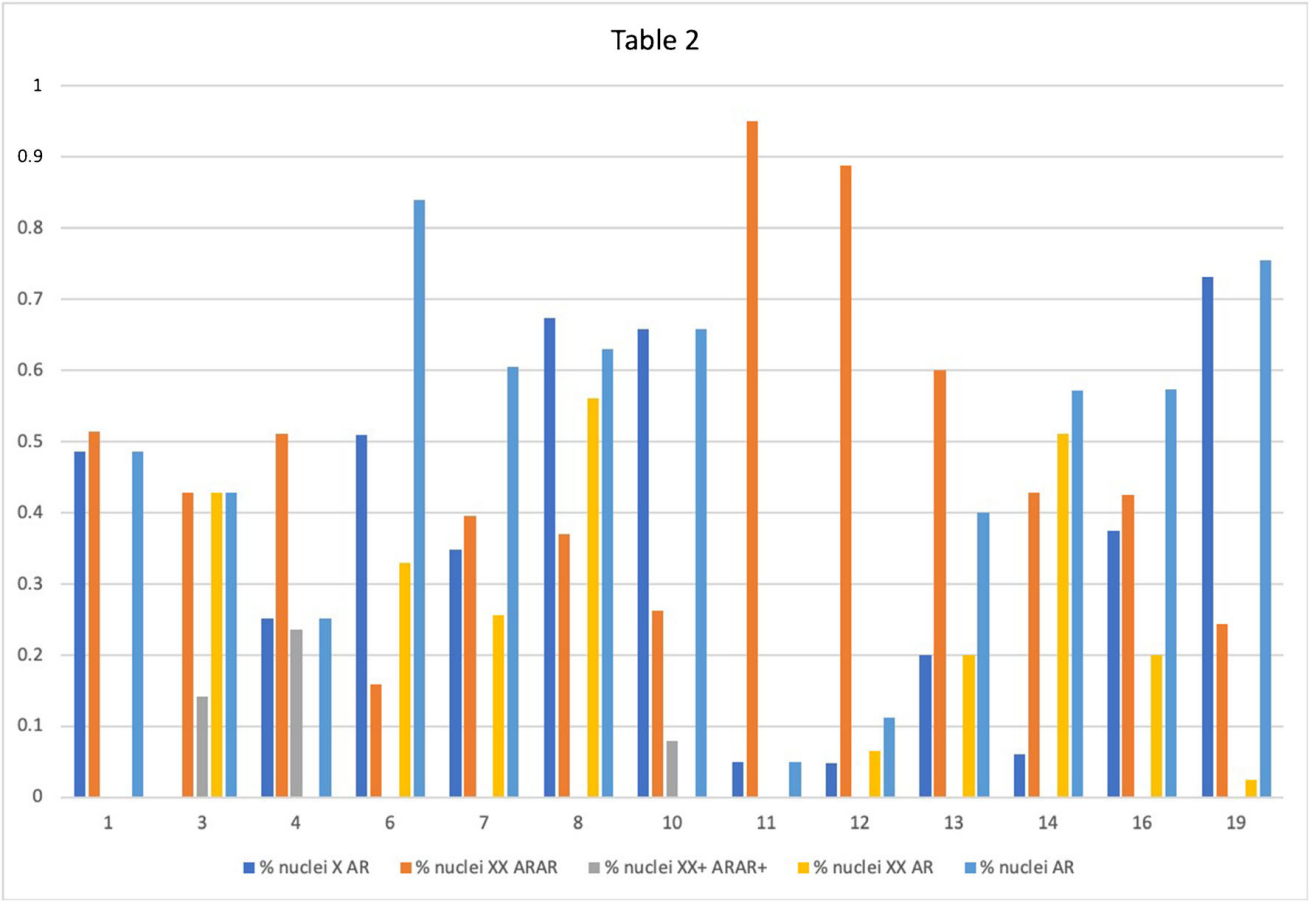
Graphic illustrating the AR loss and X chromosome aneuploidy summarized in Table 2

**Fig. 6:**
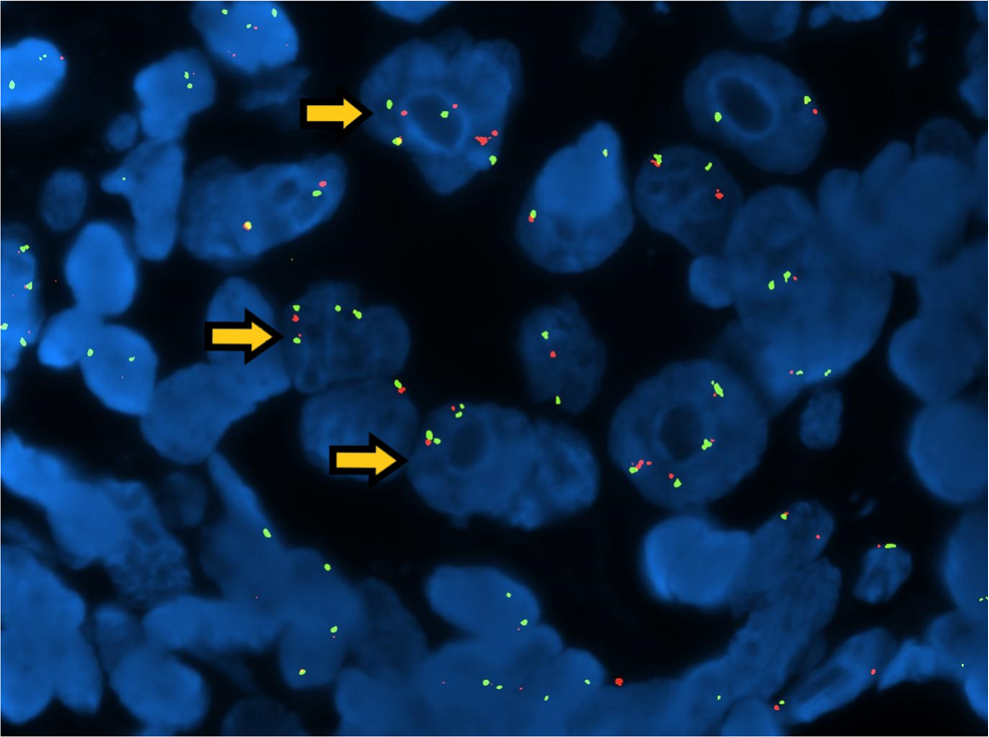
Fish analysis: AR is stained red and X chromosome is green. AR loss is evident as the number of green signals is higher than the red ones (arrows)

**Fig. 7:**
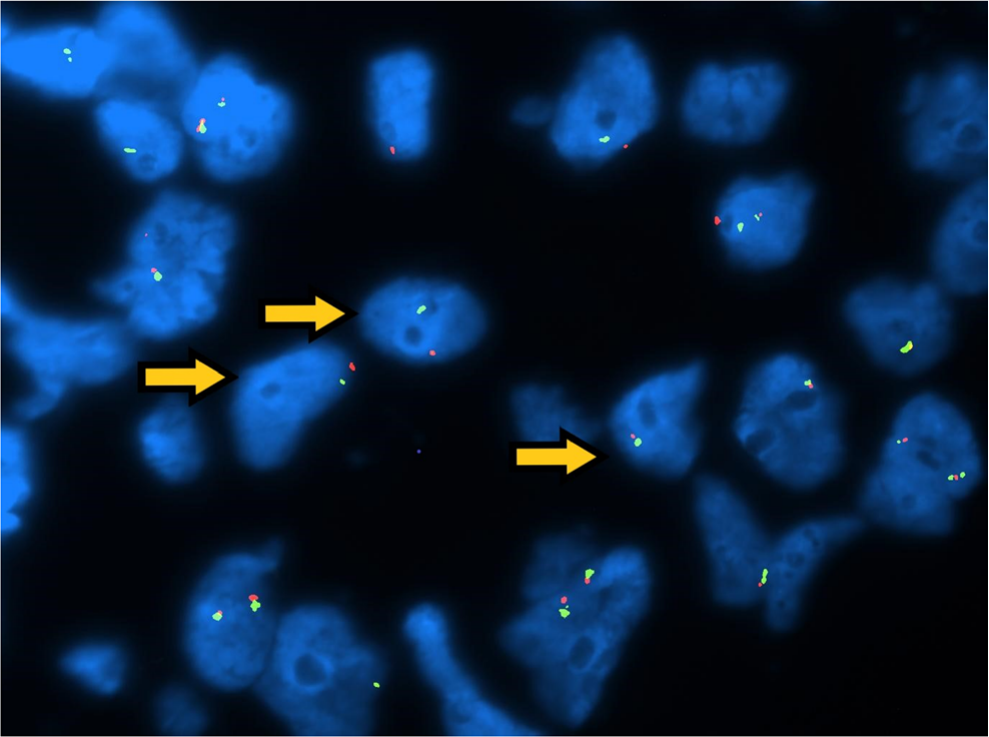
Fish analysis: In the present field, both green and red signals are reduced, demonstrating X chromosome and AR monosomy (arrows)

X chromosome copy number variation was observed in 13 of 13 tested tumours. Specifically, all except one (case 3) showed a proportion of neoplastic cells with loss of one X chromosome copy (monosomy) (Fig. 6). The percentage of neoplastic cells displaying X chromosome monosomy (Fig. 7) ranged from 4.63 to 73.10% (average 31.3%).

**Fig. 8:**
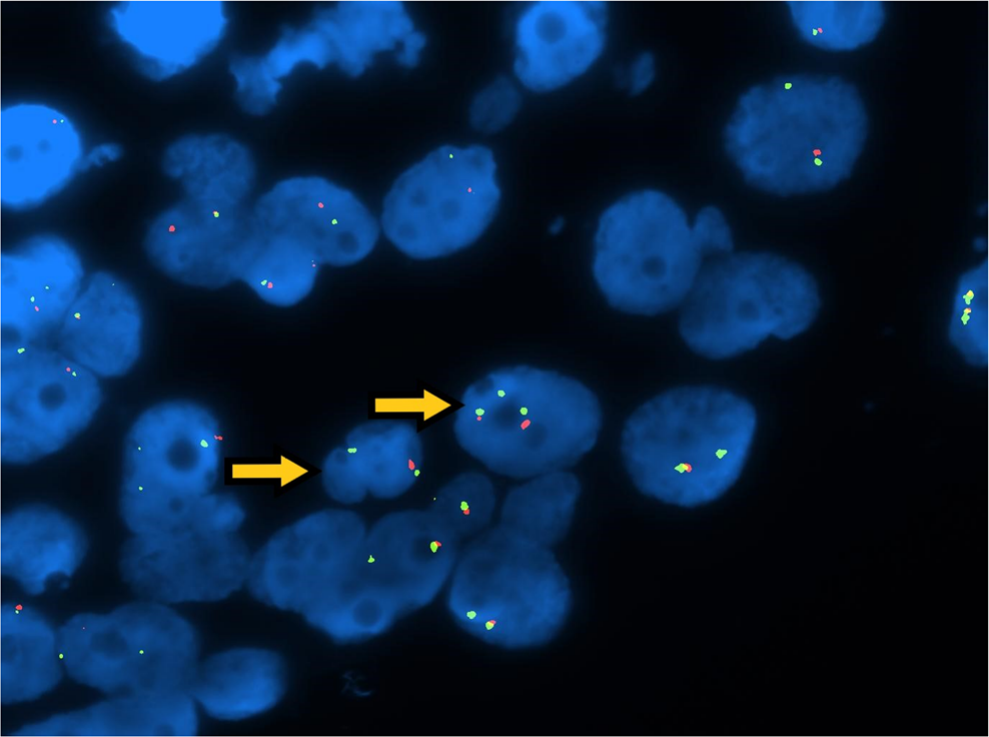
Fish analysis: some nuclei show X chromosome number increase, as three green signals are seen, not paralleled by AR polysomy as the number of red signals is lower (arrows)

In three tumours (cases 3, 4 and 10), a neoplastic population with an additional X chromosome copy (polysomy) (Fig. 8) was identified. X chromosome polysomy affected 7.89% (case 10) to 23.66% (case 3) of the neoplastic population.

Not all additional copies of the X chromosome carried the AR gene leading to AR gene relative deletion. AR gene deletion was detected in 9/13 cases and affected from 2.43 to 56.17% (average 28.62%) of the neoplastic cells.

Overall, AR gene loss (due to either X chromosome monosomy or AR gene deletion) was observed in all 13 cases tested. The neoplastic cell population showing loss of one AR gene copy varied from 5 to 84% (mean 48.93%). Minimal differences were observed between HER2-positive and HER2-negative cases (Table 3). HER2-positive cases showed a tendency towards a higher level of X chromosome monosomy.

**Table 3:** Comparison between FISH results according to HER2 status

	Number of cases	% X AR single copy
HER 2 FISH pos	6	57.71%
HER 2 FISH neg	7	46.54%

*FISH pos*, amplified on FISH analysis; *FISH neg*, not amplified on FISH analysis; *% AR single copy*, average percentage of neoplastic cells with a single AR gene copy number

In situ duct carcinoma, present in 1 case, showed AR gene monosomy in 23.8% (case 10) of the neoplastic cells.

Non-neoplastic, non-apocrine cells present around the tumour showed AR monosomy in two cases, in 15.73% and 4.17% of the cell population respectively. The related tumours showed AR monosomy in 25.19% and 57.14% of the neoplastic cells (cases 4 and 14).

### Methylation analysis

All 20 tumours were tested, but informative DNA was obtained in 9 cases only due to DNA over-fixation problems. The methylation status of the *AR*, *FLNA* and *UXT* genes, and of the MAGE family genes (*MAGEA1*, *MAGEA2*, *MAGEA3*, *MAGEA9*, *MAGEA11*, *MAGEC1*, *MAGEC2*), all present on the X chromosome, was evaluated. *MAGEG1*, a member of the *MAGE* family that maps to chromosome 15, served as a control as it is not affected by X chromosome inactivation in females. It was consistently non-methylated, as expected, as chromosome 15 is not subject to lyonization.

In all tumours, the tested genes showed variation in methylation status, with respect to the methylation condition of a single chromosome (50% of methylation).

The AR gene showed a mean methylation value in the CpG islands lower than 50% in all but one case (case 20) (Table 2 in Supplementary files shows the data related to the methylation of each CpG island of AR).

*MAGE* family members that mapped on chromosome X were hypermethylated, with methylation values varying from 42.8 to 100% (average 81.43%) (the mean methylation values obtained for each gene are shown in Table 3 Supplementary files).

*FLNA* gene displayed a variable methylation pattern ranging from 0 to 70% (Table 4, Supplementary files).

**Table 4:** Correlation between FISH results and Methylation profile

Case n.	AR ihc	AR monosomy	AR mean meth	FLNA mean meth	UXT mean meth	MAGEA1 mean meth	MAGEA2 mean meth	MAGEA3 mean meth	MAGEA9 mean meth	MAGEA 11 mean meth	MAGEC1 mean meth	MAGEC2 mean meth
1	+++	48.57	0.26	0	0.25	0.7	0.868	0.6786	0.9438	0.899	0.619	0.625
4	+++	25.19	0.22	0.49	0.32	0.5	0.8574	0.819	0.9246	0.5429	0.7143	0.8542
6	+++	84	0.07	0.06	0.24	0.9167	0.88	0.9143	0.8167	1.000	0.8218	0.8981
7	++	60.46	0.41	0.43	0.38	0.641	0.8748	0.9152	0.9099	0.8205	0.7651	0.8045
11	+++	5.00	0.5	0.46	0.16	0.8333	0.8836	0.8929	1.000	0.9109	0.4286	0.8438
12	+++	11.21	0.47	0.70	0.50	0.9167	0.9139	0.8929	0.8571	0.6842	0.7256	0.9135

*AR ihc*, androgen receptor on immunohistochemistry; *AR monosomy*, percentage of neoplastic cells with AR monosomy

*UXT* gene also displayed a variable methylation pattern ranging from 4 to 50% (Table 5, Supplementary files).

### Correlation between FISH results and methylation profile (Table 4, Fig. 9)

A complete immunohistochemical and molecular profile was obtained in 6 tumours. In all 6 tumours, AR immunohistochemical expression was high (positivity in > 95% of the neoplastic cells in 5/6 cases) in spite of X chromosome monosomy and AR gene loss in a variable percentage of neoplastic cells. All cases showed variable levels of hypomethylation of the tested genes. Even if a statistically significant correlation was not detected, methylation levels were lower in cases with higher AR gene loss, thus suggesting that the residual AR gene was transcriptionally active.

**Fig. 9:**
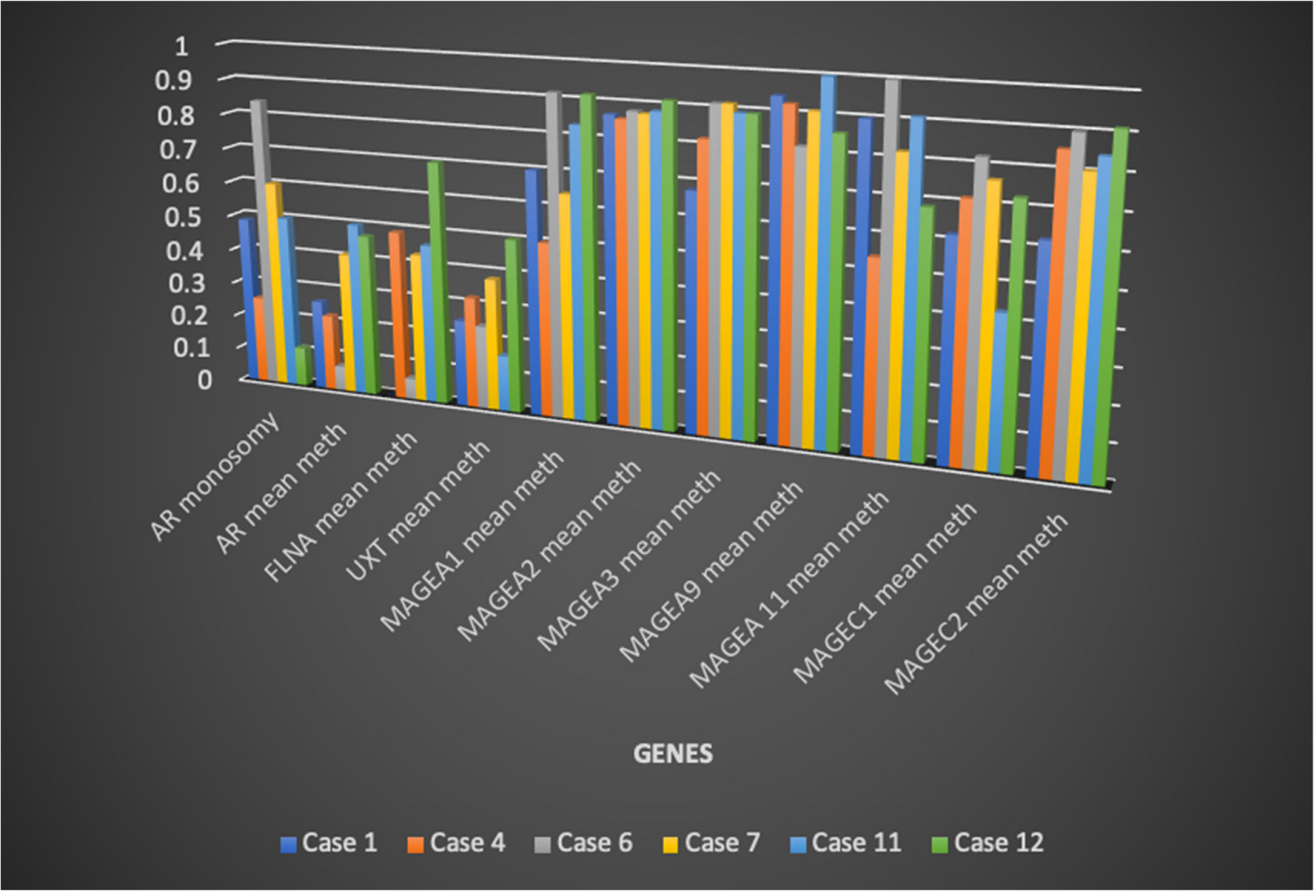
Graphic illustration of the correlation between AR, X chromosome and methylation, summarized in Table 4

## Discussion

AR is a targetable molecule, widely used in the treatment of prostatic cancer [4]. Prostatic cancer may develop AR deprivation resistance [4] for several reasons including AR amplification [18, 19]. More recently, anti-AR therapy has been tested in males with breast cancer and in small series of female breast cancer patients [4] with promising but not always consistent results. Most studies focusing on anti-AR therapy in breast cancer are based on AR expression in neoplastic cells, evaluated with immunohistochemistry. A single functioning AR gene may produce immunohistochemically detectable AR. The amount of AR protein expression depends on AR gene transcription activity. Therefore, AR gene copy number variations, as well as AR gene regulator methylation status, may have a strong influence on AR protein expression.

In the present series, almost all CADs tumours showed X chromosome aneusomy, mostly due to monosomy. AR monosomy was also present, even if at lower levels, in in situ carcinoma.

All tumours showed AR protein expression on immunohistochemistry, with greater than 95% of the neoplastic population staining positively in 19/20 cases. The AR protein expression can be explained by the hypomethylation status of the *AR* gene and its regulators. Unfortunately, the correlation between FISH and methylation results was possible in 6 cases only. In these cases, it was shown that *AR* gene monosomy was related to the hypomethylation status of the gene and most of its regulators.

These results lead to two considerations: X chromosome aneusomy can play a role in the neoplastic transformation of mammary epithelium and may regulate therapeutic response to molecules for anti-AR therapy.

The hypothesis of AR aneusomy role in the neoplastic transformation is supported by the fact that in situ duct carcinoma present in the FISH sections demonstrated *AR* gene monosomy. Furthermore, in 2 cases, *AR* monosomy was observed in the normal ductal cells surrounding the invasive carcinoma. X chromosome aneusomy has rarely been studied in breast carcinogenesis. Persons et al. [20] studied a series of 55 breast carcinomas of no special type, applying FISH analysis in order to detect chromosome copy number variations. X chromosome loss was present in a minority of cases and was related to lymph node metastases and tumour grade. X chromosome aneusomy and related *AR* gene copy number aberrations have been demonstrated in male breast cancer. However, male breast cancer, in contrast with the female CADs here studied, showed mainly X chromosome polysomy and related *AR* gene copy number gain [10, 11].

The second consideration focuses on the possible role of AR as a therapeutic target. Student et al. [4] reviewed the application of anti-AR therapy in different diseases, including ER- and PR-negative breast cancers. Accordingly, promising results are being published based on response to latest-generation anti-AR hormonal drugs.

All the studies published to date focus on AR expression evaluated by immunohistochemistry. The data here shown demonstrate a great variability of *AR* gene status paralleled by a great variability of *AR* and its regulator methylation status. In most of the studied tumours, the *AR* gene was monosomic and its regulators showed a variable methylation status, with a prevalence of hypomethylation. These conditions lead us to hypothesize that the monosomic *AR* gene is transcriptionally active, explaining why most of the neoplastic cells show AR positivity on immunohistochemistry. Nevertheless, differences in *AR* gene status could lead to quantitative differences in AR protein production. It is plausible to hypothesize that AR protein can be reduced in *AR* monosomic neoplastic cells.

In patients with prostatic cancer, it has been demonstrated that *AR* gene polysomy may lead to castration therapy resistance [18, 19]. Therefore, anti-AR therapy might be more effective in those patients with lower AR protein production.

The present work has some limitations, including the low case number, inability to perform quantitative evaluation of AR protein with western blot analysis due to the lack of freshly fixed tissue and some compromised FISH and DNA studies due to fixation issues.

Despite these limitations, the data here shown demonstrate that in breast CADs even if AR immunohistochemical expression is high, *AR* transcriptional status may be quite variable. This variability may have an impact on response to AR deprivation therapy.

## Supplementary Information

ESM 1 (DOCX 18 kb)

## Data Availability

All data generated or analysed during this study are included in this published article (and its supplementary information files).
