# Supplementary material for: Chromosome X aneusomy and androgen receptor gene copy number aberrations in apocrine carcinoma of the breast
Source: Virchows Arch. 2021 Feb 3;479(2):345–54. doi: 10.1007/s00428-021-03028-2 (PMC8364532; doi:10.1007/s00428-021-03028-2)
Supplement: Supplementary file 1 — (DOCX 18 kb) [file 428_2021_3028_MOESM1_ESM.docx]

Table 1s: Genetic coordinates of investigated regions

| *Gene* | Description | Primer Forward | Primer Reverse | Reference |
| --- | --- | --- | --- | --- |
| **AR** | *Androgen Receptor* | GAGGAGTTTTTTAGAATTTGTTTTAGAG | AAAAACCATCCTCACCCTACTACTAC | di Oto at al. |
| **MAGEA1** | MAGE family member A1 | GATTTAGGTTTTGTGAGGAGGTAAG | CAAAAATCTAAAAACAACCCAAACTA | This work |
| **MAGEA2** | MAGE family member A2 | TTTTTGTYGTGAATTTAGGGAAG | AATAAAACCCRCCTCAATCC | This work |
| **MAGEA3** | MAGE family member A3 | ATTTTTGTTYGGAATTTAGGG | TCAATCCTCCCTCAACRTCT | This work |
| **MAGEA9** | MAGE family member A9 | GGGTGTAGGAAAATTATTTGGAGTAT | CCCAAAACCTCTTCATAAAAAAATA | This work |
| **MAGEA11** | MAGE family member A11 | GGGAGGATTGAGGTATTTTTATGAT | ACTTCCCTAAATTTACAACAAAAAC | This work |
| **MAGEC1** | MAGE family member C1 | TAGTAGGGTTTAGGGAGTGAGTAGAAA | TCAAAATTAATCAAAACTAACAACCC | This work |
| **MAGEC2** | MAGE family member C2 | TGTTGGATTTTATTATTTATATTTTTGTTG | AAACTTCCTCCTCTTCCTCATCTATA | This work |
| **MAGEG1** | MAGE family member G1 | GGTTAGGTYGAGAGGGATAGAGA | AAAAACCCTACRACCCCTAC | This work |
| **UXT** | Ubiquitously expressed prefoldin like chaperone | GTTTGGGTGTTTTTGGGTGGT | TCCAATTTAACCTCACACACAATTCAT | This work |
| **FLNA** | Filamin A | TGGAAGAAGATTTAGTAGAATATTTTTA | CTTCTAACTAAACACCTCCAACAAC | This work |

Table 1s continues

| ***Gene*** | **Map** | **ENSEMBL** | **Position** | **UCSC h38 coordinates** | **Amplicon length** | **Position respect to TSS** | **N of interrogated CpG** |
| --- | --- | --- | --- | --- | --- | --- | --- |
| **AR** | Xq11-12 | ENSG00000169083 | Exon 1 | ChrX + strand: 67545205-67545435 | 231 | 1169 | 9 |
| **MAGEA1** | Xq28 | ENST00000356661.7 | Exon 1 | ChrX + strand: 153179341-153179499 | 159 | -3046 | 6 |
| **MAGEA2** | Xq28 | ENSG00000268606 | Exon 1 | ChrX - strand: 152753752-152753935; ChromX + strand: 152714535-152714718 | 184 | -57 | 16 |
| **MAGEA3** | Xq28 | ENST00000598245.2 | Promoter | ChrX + strand: 152698673-152698851 | 180 | -2156 | 16 |
| **MAGEA9** | Xq28 | ENST00000243314.5 | Promoter | ChrX + strand: 149786956-149787136 | 181 | +745 | 5 |
| **MAGEA11** | Xq28 | ENSG00000185247 | intron1-2 | ChrX + strand: 149711859-149712030 | 172 | 22883 | 15 |
| **MAGEC1** | Xq27.2 | ENSG00000155495 | Promoter | ChrX + strand: 141903673-141903833 | 161 | -1400 | 7 |
| **MAGEC2** | Xq27.2 | ENSG00000046774 | Exon 3 | ChrX - strand: 142203870-142204050 | 181 | -63 | 8 |
| **MAGEG1** | 15q13.1 | ENST00000332303.6 | Exon 1 | Chr15 - strand: 29269516-29269669 | 154 | +36 | 18 |
| **UXT** | Xp11.3 | ENSG00000126756 | Exon 1 | ChrX + strand: 47658973-47659103 | 130 | +78 | 6 |
| **FLNA** | Xq28 | ENST00000369850.10 | Exon 2 | ChrX - strand: 154370985-154371125 | 150 | +146 | 12 |

Table 2s. Mehtylation of each CpG in AR gene (genome coordinates are based on hg38)

| CpG  Case | 67545233 | 67545237 | 67545239 | 67545256 | 67545278 | 67545280 | 67545284 | 67545296 | 67545299 | median |
| --- | --- | --- | --- | --- | --- | --- | --- | --- | --- | --- |
| **1** | 0,274 | 0,265 | 0,265 | 0,209 | 0,343 | 0,275 | 0,343 | 0,346 | 0,023 | 0,26 |
| **4** | 0,243 | 0,245 | 0,245 | 0,123 | 0,208 | 0,208 | 0,245 | 0,236 | 0,208 | 0,22 |
| **4** | 0,032 | 0,032 | 0,032 | 0,016 | 0,095 | 0,095 | 0,079 | 0,111 | 0,016 | 0,06 |
| **6** | 0,102 | 0,075 | 0,101 | 0,068 | 0,026 | 0,059 | 0,084 | 0,081 | 0 | 0,07 |
| **7** | 0,478 | 0,394 | 0,446 | 0,19 | 0,365 | 0,398 | 0,53 | 0,498 | 0,373 | 0,41 |
| **11** | 0,589 | 0,633 | 0,581 | 0,317 | 0,512 | 0,496 | 0,512 | 0,557 | 0,309 | 0,5 |
| **12** | 0,558 | 0,519 | 0,551 | 0,372 | 0,436 | 0,429 | 0,615 | 0,526 | 0,231 | 0,47 |
| **16** | 0,431 | 0,48 | 0,48 | 0,011 | 0,112 | 0,301 | 0,407 | 0,364 | 0,349 | 0,32 |
| **20** | 1 | 1 | 1 | 0,857 | 0,857 | 0,992 | 1 | 1 | 0 | 0,85 |

Table 3s. Median methylation values of each *MAGE* family.

| Case | *MAGEA1* | *MAGEA2* | *MAGEA3* | *MAGEA9* | *MAGEA11* | *MACEC1* | *MAGEC2* |
| --- | --- | --- | --- | --- | --- | --- | --- |
| **1** | na | 0,8680 | 0,6786 | 0,9438 | na | 0,6190 | 0,6250 |
| **4** | 0,5000 | 0,8574 | 0,8190 | 0,9246 | 0,5429 | 0,7143 | 0,8542 |
| **5** | 0,5694 | 0,8528 | 0,7476 | 0,8940 | 0,6741 | na | 0,6362 |
| **6** | 0,9167 | 0,8800 | 0,9143 | 0,8167 | 1,0000 | 0,8218 | 0,8981 |
| **7** | 0,6410 | 0,8748 | 0,9152 | 0,9099 | 0,8205 | 0,7651 | 0,8045 |
| **11** | 0,8333 | 0,8836 | 0,8929 | 1,0000 | 0,9109 | 0,4286 | 0,8438 |
| **12** | 0,9167 | 0,9139 | 0,8929 | 0,8571 | 0,6842 | 0,7256 | 0,9135 |
| **16** | na | 0,7151 | 0,8980 | 0,8596 | na | 0,8367 | 0,9063 |
| **20** | na | 0,8929 | 0,9286 | 0,9520 | na | 0,5714 | 0,7500 |
